# Exposure of children to smoke in clay figures craft in Caruaru, state of Pernambuco, Brazil

**DOI:** 10.47626/1679-4435-2023-844

**Published:** 2023-04-18

**Authors:** Géssica Coelho Ambrozim, Lorenzo Durão Cápua, Luiz Eduardo Lira Perazzo, Vinícius Zidane Silva Nascimento, Juliana Martins Barbosa da Silva Costa

**Affiliations:** 1 Núcleo de Ciências da Vida, Universidade Federal de Pernambuco, Caruaru, PE, Brazil

**Keywords:** child, hypersensitivity, respiratory tract diseases, biomass, smoke, criança, hipersensibilidade, doenças respiratórias, biomassa, fumaça

## Abstract

**Introduction:**

Alto do Moura, a neighborhood located in the city of Caruaru, state of Pernambuco, Brazil, is known by the production of figurative art in clay, which uses wood as the main fuel in its finishing process. Chronic exposure to toxic gases released in combustion can trigger respiratory atopies.

**Objectives:**

To identify children with respiratory atopies accompanied by the Alto do Moura Family Health Unit and the spatial distribution of furnaces used in the burning of figurative art in clay.

**Methods:**

This was an exploratory, observational, descriptive, cross-sectional study analyzing 596 medical records of children with respiratory atopies living in the aforementioned neighborhood from July 2018 to October 2020. Fifty-two children aged 2 to 10 years were identified. A sociodemographic questionnaire was applied, and the location of furnaces, source of smoke, was mapped. Data were collected using the HC Maps^®^ application, which stores and generates an electronic spreadsheet for analysis. The prevalence of respiratory atopies and the average distance between children’s homes and furnaces were calculated.

**Results:**

A prevalence of respiratory atopies of 8.6% was found in the population studied. Allergic rhinitis was the most common diagnosis, followed by asthma. School-age children were the most affected group, and the average distance between children’s homes and furnaces was 76.8 meters.

**Conclusions:**

The presence of environmental pollution resulting from burning wood for making figurative art in clay may be contributing to the occurrence of respiratory atopies in children. Preventive measures, such as using exhaust fans, opening windows, and increasing ventilation, should be encouraged.

## Abstract (pt)

**Introdução:**

O bairro Alto do Moura, situado na cidade de Caruaru, no estado de Pernambuco, é conhecido pela produção de arte figurativa em barro, que utiliza madeira como principal combustível em seu processo de finalização. A exposição crônica aos gases tóxicos liberados na combustão pode desencadear atopias respiratórias.

**Objetivos:**

Identificar as crianças com atopias respiratórias atendidas na Unidade de Saúde da Família do Alto do Moura e a distribuição espacial das fornalhas utilizadas na queima da arte figurativa em barro.

**Métodos:**

Tratou-se de estudo exploratório, observacional-descritivo, transversal, mediante análise de 596 prontuários de crianças com atopias respiratórias residentes no bairro no período de julho de 2018 a outubro de 2020. Foram identificadas 52 crianças, na faixa etária de 2 a 10 anos. Foi aplicado formulário sociodemográfico, e a localização das fornalhas, fonte da fumaça, foi mapeada. Realizou-se a coleta dos dados com o aplicativo HC Maps^®^, que os armazena e gera planilha eletrônica para análise. Calculou-se a prevalência das atopias respiratórias e a distância média entre as residências das crianças e as fornalhas.

**Resultados:**

Encontrou-se uma prevalência de atopias respiratórias de 8,6% na população estudada. A rinite alérgica foi o diagnóstico mais comum, seguida de asma. Os escolares foram os mais acometidos, e a distância média entre as residências das crianças e a localização das fornalhas foi de 76,8 metros.

**Conclusões:**

A presença de poluição ambiental decorrente da queima da madeira para confecção da arte figurativa em barro pode estar contribuindo para a ocorrência de atopias respiratórias em crianças. Medidas preventivas, como uso de exaustores, abertura de janelas e melhoria na ventilação, devem ser estimuladas.

## INTRODUCTION

The city of Caruaru, located in the microregion of Vale do Ipojuca, mesoregion of Agreste in the state of Pernambuco, Brazil, is internationally known for great production of figurative art, especially Alto do Moura, a neighborhood located in the West region of the municipality, in an area of transition between rural and urban environments. A fundamental process for the production of clay figures is burning. To that end, wood is the main fuel used to create heat, releasing carbon monoxide (CO), methane, volatile organic compounds (VOC), nitrogen oxides (NOx), polycyclic aromatic hydrocarbons (PAH), and particulate matter (PM).^1^ As a consequence, exposure to these toxics may cause coughing and mucosa and throat irritation, also to leading to acute respiratory tract inflammation and reduced pulmonary function.^2^ In Caruaru, the prevalence of asthma is approximately 18%,^3^ with higher rates of symptoms in children from families that use open fires compared to those with improved stoves with chimneys.^1^

The International Study of Asthma and Allergies in Childhood (ISAAC) observed a prevalence of nasal symptoms of approximately 37.2% among children and adolescents in Brazil, ranging from 26.3 to 49.9%.^3^ Furthermore, Sakano et al.^4^ describe rhinitis as an inflammation and/or dysfunction of the nasal mucosa characterized mainly by nasal obstruction, anterior and posterior rhinorrhea, sneezing, nasal pruritus, and hyposmia, which may occur for 2 or more days for an average of 1 hour per day.

Atopy is considered an exacerbated immunological response mediated by immunoglobulin E (IgE) antibodies, i.e., by a type I hypersensitivity mechanism. Allergy, in turn, is defined as an exaggerated immunological reaction to an antigen, with no specific immunological mechanism. Triggering factors are classified into three categories: aeroallergens, pollutants, and irritating agents.^4^ Considering the focus of this study, i.e., pollutants, triggers include cigarette smoke, gases emitted by wood burning (especially NOx), ozone gas, and sulfur dioxide.^4^ Allergy symptoms favor mouth breathing, which causes a series of orofacial changes, such as long and thin face, underdeveloped maxilla, and ogival palate, in addition to thoracic asymmetry and behavioral disorders like restlessness, irritation, and agitated sleep.^1^ These symptoms may affect children’s speech and concentration, negatively interfering with school performance. Moreover, changes in the growth of skull and dental arch were observed, with the latter causing problems related to child nutrition.^1^

Hence, this study aimed to determine the epidemiological profile of preschool and school-age children with respiratory atopy living in Alto do Moura neighborhood and to explore possible relationships between exposure to smoke from furnaces used to produce figurative art in clay and occurrence of atopy.

## METHODS

An exploratory, observational, descriptive, cross-sectional study was conducted. Research took place from July 2018 to October 2020 with the population assigned to the Family Health Unit, considering the geographical region of Alto do Moura, a neighborhood in Caruaru, state of Pernambuco, Brazil, to evaluate 596 medical records.

In this analysis, research participants were selected according to the following inclusion criteria: preschool children (2 to 4 years) or school-age children (5 to 10 years) belonging to the population assigned to Alto do Moura Family Health Unit and who were affected by respiratory atopies described in the following Chapter X codes of the International Classification of Diseases -10th revision (ICD-10): J30 (vasomotor and allergic rhinitis), J31 (chronic rhinitis, nasopharyngitis and pharyngitis), J32 (chronic sinusitis), J39 (other diseases of upper respiratory tract), J45 (asthma) and T78.4 (unspecified allergy), as long as the child presented respiratory symptoms, or by the following codes of the R (respiratory system) chapter of the International Classification of Primary Care (ICPC-2): R97 (allergic rhinitis), R75 (chronic/acute sinusitis) and R96 (asthma). Children outside the above age group or who did not suffer any of the aforementioned diseases were excluded from the study.

Home visits were made to the selected children to apply a form with questions on participants’ sociodemographic profile and on the presence of risk factors that contributed to the development of respiratory atopies. Furthermore, at the time of the visit, household location was registered in the HC Maps^®^ geolocation application, leased for use through a partnership with Instituto Aggeu Magalhães (IAM), of Oswaldo Cruz Foundation (Fundação Oswaldo Cruz, Fiocruz). Three attempts of contact with children’s guardians were made, and, if they were not found at home, children were excluded from the research. Data analyzed in the questionnaire were kinship bond between guardian and child, race, age, sex, number of household members, presence of smokers, presence of wood-burning stove, use of treatment, and family history of respiratory atopy. These data were compiled in the HC Maps^®^ software, generating a database on the cloud, and then transcribed to a Microsoft Excel^®^ spreadsheet for analysis. This made it possible to perform the statistical calculation of the prevalence of respiratory atopies, using the number of atopic children identified in the research as the numerator and the total number of medical records as the denominator.

Furnaces present in the territory were identified in a partnership with community health workers, and the location of these furnaces was registered using the HC Maps^®^ application. Later, the maps obtained from the system showing the location of atopic children and of furnaces were overlapped using the open web-based software Google Maps^®^. Subsequently, the distance of each child with atopy and the nearest furnace was measured on the map, using the electronic tool available in the application, and converted into meters by a predefined scale. Finally, measures of central tendency (arithmetic mean, mode, and median) were calculated in order to detail the analysis.

The research project was approved by the Research Ethics Committee of Universidade Federal de Pernambuco, under registration number 3.219.387.

## RESULTS

Of the 596 medical records analyzed, 51 children with the ICD-10/ICPC 2 codes included in the research, yielding a prevalence of respiratory atopies of 8.56% in the region. The most observed diagnoses observed in the study population were vasomotor and allergic rhinitis (ICD-10 J30), asthma (ICD-10 J45), and unspecified allergy (ICD-10 T78.4) (Table 1). Among the children selected, 56.86% were female, and 43.14% were male; furthermore, 31.37% were preschool children, and 68.63% were school-age children.

**Table 1:** Distribution of atopic children based on the International Classification of Diseases or on the International Classification of Primary Care

ICD-10	n	%
J30	17	33.33
J31	0	0.00
J32	1	1.96
J39	2	3.92
J45	22	43.14
T78.4	8	15.69
		
ICPC 2	n	%
R75	0	0.00
R96	0	0.00
R97	1	1.96

ICD-10 = International Classification of Diseases, 10th revision; ICPC 2 = International Classification of Primary Care.

At the time of home visits to conduct the interviews, three children did not live in the neighborhood anymore, and 17 guardians were not found after three attempts, being thus excluded from the study. Therefore, 31 guardians were interviewed, most of which (87.10%) were children’s mothers. The predominant profile of the sample was characterized by brown (mixed-race), school-age (from 5 to 12 years), and female children.

With regard to the number of household members, 25.81% of households had from one to three residents, and 74.19% of them had from four to six residents. Additionally, 87.10% of children did not live with a smoker in the household, and only 6.45% of households had a wood-burning stove. Furthermore, only 25.81% of children received treatment for their respiratory atopy. Family history of a 1^st^-degree relative with this disease was present in 51.61% of individuals (Table 2).

**Table 2:** Sociodemographic characteristics and environmental exposure variables of the children studied

Variables	n	%
Kinship bond with the child		
Mother	27	87.10
Father	3	9.68
Grandmother	1	3.22
Ethnicity		
Black	2	6.45
Brown	17	54.84
White	12	38.71
Yellow	0	0.00
Indigenous	0	0.00
Age (years)		
Preschool children (2 to 4)	13	41.94
Schoolchildren (5 to 10)	18	58.06
Sex		
Female	20	64.52
Male	11	35.48
Number of household members (people)		
1 to 3	8	25.81
4 to 6	23	74.19
≥ 7	0	0.00
Smoker in the household		
Yes	4	12.90
No	27	87.10
Do not know	0	0.00
Wood-burning stove		
Yes	2	6.45
No	29	93.55
Do not know	0	0.00
Treatment for atopy		
Yes	8	25.81
No	22	70.97
Do not know	1	3.22
Family history of atopy in a 1^st^-degree relative		
Yes	16	51.61
No	15	48.39
Do not known	0	0.00

**Figure 1:**
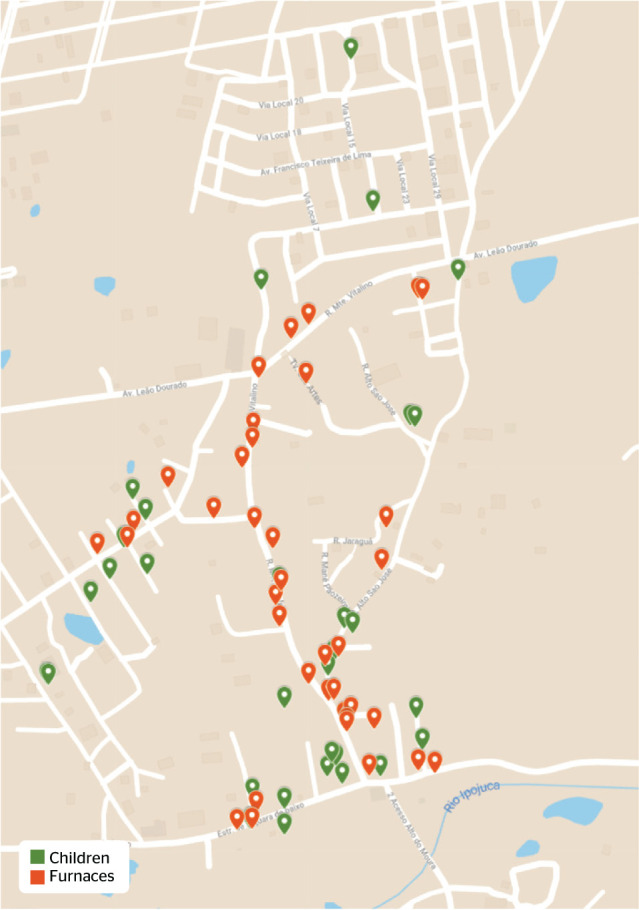
Geographic distribution of children and furnaces in Alto do Moura neighborhood, Caruaru, state of Pernambuco, Brazil.

Thirty-seven furnaces were cataloged in the territory using the HC Maps^®^ application, none of which had an air filter at the time of data collection. The distance between location of furnaces and location of atopic children is presented in Figure 1. The highest distance between a child with atopy and the nearest furnace was 390 meters, whereas the lowest one was 5 meters. Measures of central tendency calculated from the minimum distances between each child and the nearest furnace were average of 76.8 meters, median of 50 meters, and mode of 45 meters.

## DISCUSSION

In general, atopic children from the neighborhood analyzed were aged from 5 to 10 years, were female, and were diagnosed with bronchial asthma, followed by allergic rhinitis. The prevalence of respiratory atopy found in the present study was 8.56%. Similarly, a cross-sectional study by Riguera et al.^5^ assessed the prevalence of asthma and rhinitis symptoms according to the protocol from the International Study of Asthma and Allergies in Childhood (ISAAC) study in children aged from 10 to 14 years living in the municipality of Monte Aprazível, state of São Paulo, Brazil, whose economy is strongly related to sugarcane production and processing. As a result, the authors found that the prevalence of asthma in the municipality (10.6%) was below the national average (11.8%), whereas the prevalence of rhinitis symptoms (33.2%) was above the Brazilian average (30%), both described by the ISAAC. It was also observed that, during the sugarcane harvest season, a period of increased outbreaks of sugarcane burning, there is a raise in the number of exacerbations of respiratory symptoms and of asthma and rhinitis episodes. Conversely, in a study conducted in the municipality of Alta Floresta, state of Mato Grosso, Brazil, located in an area with a high concentration of burning outbreaks in the Amazon region, with average estimated concentration levels of fine PM of 40.6 µg/m^3^, de Farias et al.^6^ observed a prevalence of asthma of 21.4% among school-age children, similar to the Brazilian average, but one of the highest rates in Latin America.

Our analysis also revealed that 51.61% of children had a family history of respiratory atopy in 1^st^- degree relatives. The genetic component itself does not explain the development of asthma, despite increasing its risk, especially in the case of maternal asthma history.^7^ For allergic rhinitis, positive diagnosis in one of the parents doubles the risk of children developing the disease. Furthermore, rhinitis itself significantly increases the likelihood of developing asthma by 40%, with the common concomitant occurrence of atopic eczema.^8^

Norbäck et al.^9^ showed that warmer climate, exposure to outdoor nitrogen dioxide and PMs < 10 µm, tobacco smoke, gas cooking, burning biomass, incense and mosquito coils were associated with increased risk of asthma, wheeze, rhinitis, and eczema among pre-school children in China. In a study conducted with 358 children living in the fishing port of Ibaka, Nigeria, Oloyede et al.^10^ found that those chronically exposed to wood smoke present higher rates of lung function decline. Similar to the Alto do Moura region, residents in the port area of Ibaka uses wood as the main heat source for their main occupation, which in their case is production of smoked fish.

It was observed that most furnaces identified did not have filters to reduce emission of pollutants. Despite their beneficial effect on air quality, a study observed that use of these devices did not result in improved efficacy of asthma treatment.^11^ Conversely, it was found that exposure to wood heating and tobacco smoke over 10 years increases the risk of persisting asthma and lung function decline, particularly in people with *GST* gene risk variants.^12^

In our study, only two houses (6.45%) had a wood-burning stove, and four children lived with a smoker, both of which are well documented risk factors for respiratory atopies. Therefore, air pollution resulting from burning wood may act as triggering factor of crises in the remaining cases. Strategies to minimize the effects of exposure include using exhaust fans and opening windows to increase space ventilation.^12^

We observed that 70.97% of children did not use continuous drug therapy to prevent exacerbation of their diseases. The Global Initiative for Asthma (GINA),^13^ in line with Wheatley & Togias,^8^ attest that, both for asthma and allergic rhinitis, long-term treatment is important to control symptoms and reduce disease exacerbation and risk of disease-related mortality.

A study by Weaver et al.^14^ showed a spatial correlation between PM2.5 and living at a distance of up to 5 meters from a source of biomass burning. However, variogram analysis indicated that smoke coming from this combustion may dissipate over a distance sufficiently long to affect all neighboring houses. Our study found that the closest distance between a child and the nearest furnace was 5 meters; however, average for the minimum distance between children and furnaces was 76 meters. Weaver et al.^14^ also observed that, even in houses that did not use biomass fuel in furnaces, levels of air pollutants and CO concentrations were increased. Furthermore, during wood burning, neighbor homes with no shared wall had about a 31% increase in PM2.5 and about 100-fold increase in mean CO concentrations.

## CONCLUSIONS

The presence of environmental pollution resulting from burning wood for making figurative art in clay may be contributing to the occurrence of respiratory atopies in children living in Alto do Moura neighborhood, Caruaru, state of Pernambuco, Brazil. Preventive measures such as using exhaust fans, opening windows, and increasing ventilation should be encouraged. Further longitudinal investigations on this factor in similar environments are necessary to determine significant associations, especially studies on local air quality and evaluations of a possible mitigation of respiratory health risks with the implementation of environmental interventions, such as use of air filters in furnaces.
